# Longitudinal Changes in CT Body Composition in Patients Undergoing Surgery for Colorectal Cancer and Associations With Peri-Operative Clinicopathological Characteristics

**DOI:** 10.3389/fnut.2021.678410

**Published:** 2021-08-16

**Authors:** Ross D. Dolan, Tanvir Abbass, Wei M. J. Sim, Arwa S. Almasaudi, Ly B. Dieu, Paul G. Horgan, Stephen T. McSorley, Donald C. McMillan

**Affiliations:** Academic Unit of Surgery, School of Medicine, University of Glasgow, Glasgow Royal Infirmary, Glasgow, United Kingdom

**Keywords:** colorecal cancer, TNM, systemic inflammation, glasgow prognostic score, body composition, computer tomograph

## Abstract

There is evidence for the direct association between body composition, the magnitude of the systemic inflammatory response, and outcomes in patients with colorectal cancer. Patients with a primary operable disease with and without follow-up CT scans were examined in this study. CT scans were used to define the presence and changes in subcutaneous fat, visceral fat, skeletal muscle mass, and skeletal muscle density (SMD). In total, 804 patients had follow-up scans and 83 patients did not. Furthermore, 783 (97%) patients with follow-up scans and 60 (72%) patients without follow-up scans were alive at 1 year. Patients with follow-up scans were younger (*p* < 0.001), had a lower American Society of Anaesthesiology Grade (*p* < 0.01), underwent a laparoscopic surgery (*p* < 0.05), had a higher BMI (*p* < 0.05), a higher skeletal muscle index (SMI) (*p* < 0.01), a higher SMD (*p* < 0.01), and a better 1-year survival (*p* < 0.001). Overall only 20% of the patients showed changes in their SMI (*n* = 161) and an even lower percentage of patients showed relative changes of 10% (*n* = 82) or more. In conclusion, over the period of ~12 months, a low–skeletal muscle mass was associated with a systemic inflammatory response and was largely maintained following surgical resection.

## Introduction

Colorectal cancer (CRC) is the fourth leading cause of cancer mortality in developed countries (1). Approximately 50% of those diagnosed will die from their cancer or some other comorbid disease (2). In a similar pattern to other solid organ tumors, disease progression is associated with a progressive nutritional and functional decline resulting in poor response to treatment and poor survival (3, 4).

The relationship between weight loss and poor outcomes in patients with cancer has long been established. CT-derived body composition analysis has allowed for the specific constituent parts of cancer-related weight loss to be more formally established. Both high–CT-derived visceral and subcutaneous fat mass have been shown to be associated with increased post-operative complications and outcomes in patients with CRC (5, 6). Furthermore, more recently, it has become clear that loss of skeletal muscle mass may underlie the relationship between weight loss and poor outcomes in patients with cancer (3, 4). In particular, a low skeletal muscle index (SMI) is associated with increased length of hospital stay, with associated poorer treatment tolerance and efficacy (7, 8), worse quality of life, and increased morbidity (9). The basis of the relationship between the loss of skeletal muscle mass and poor outcomes in patients with cancer is not clear. There are a number of potential confounding factors in the relationship, including age (10), gender (11), tumor node metastasis (TNM) stage (12), comorbidity, (13, 14), and the systemic inflammatory response (15, 16).

There is evidence to a direct association between the magnitude of the systemic inflammatory response, as evidenced by systemic inflammation-based scores, such as the modified Glasgow Prognostic Score (mGPS), the neutrophil lymphocyte ratio (NLR), low–SMI, and low–skeletal muscle density (SMD) in patients with CRC (13, 17–19). However, whether these relationships are causal or merely associative is not known because only a few longitudinal and interventional studies have been published to date.

Therefore, the aim of the present longitudinal study was to delineate the relationship between longitudinal changes in CT-derived body composition, clinicopathological characteristics, and the systemic inflammatory response in patients with colorectal cancer.

## Patients and Methods

### Patients

**Figure 1:**
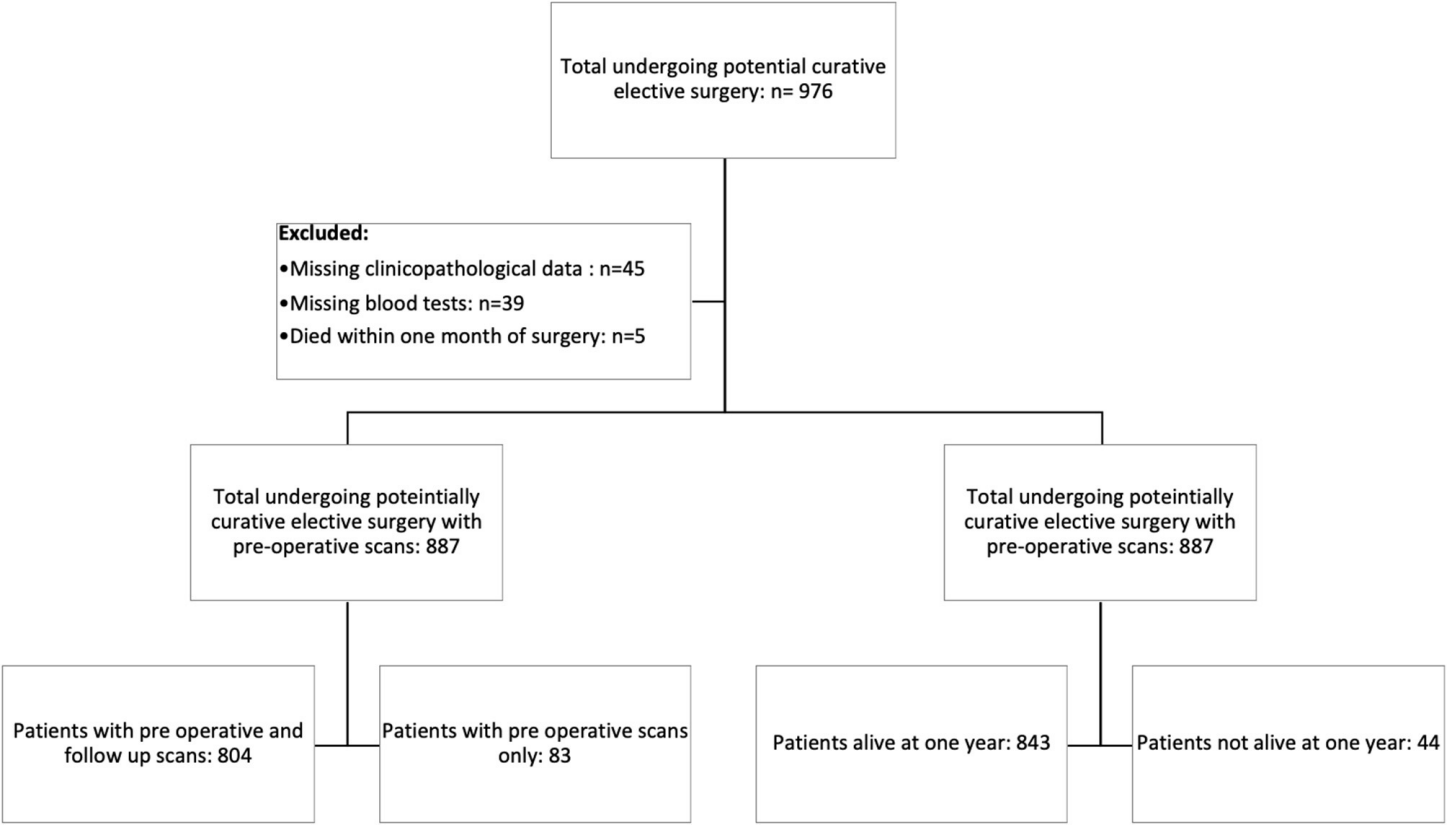
Prisma diagram of patients excluded from the study due to missing clinicopathological data, blood tests, or death within 1 month of surgery. Further subdivisions showing the number of patients with follow-up scans at 1 year and patients alive at 1 year.

Consecutive patients who underwent elective, potentially curative resections for CRC in an enhanced recovery pathway between March 2008 and June 2018 at a single center were identified from a prospectively maintained database. Patients with a preoperative scan with or without follow-up CT scans and a recorded height and weight were included in the study (Figure 1).

The patients were classified according to body mass index (BMI) as underweight/normal weight (BMI < 24.9) and overweight/obese (BMI ≥ 25.0). All tumors were staged according to TNM fifth edition (20). Preoperative hematological and biochemical markers were recorded.

The cause and date of death were confirmed with the Registrar General (Scotland) until June 1, 2018, which served as the censor date. Informed consent was obtained from patients prior to surgery. Those with metastatic CRC and those who underwent emergency surgery or palliative surgery were excluded from the study. Ethical approval was granted by the West of Scotland Research Ethics Committee, Glasgow, United Kingdom.

### Methods

Pre-operative and initial follow-up CT images were obtained at the level of the third lumbar vertebra as previously described (17) as part of their routine clinical follow-up. The median time from pre-operative scan to follow-up scan was 12 months (9–18 months). Scans with a significant movement artifact or a missing region of interest (ROI) were excluded from the study. Each image was analyzed using a free-ware program (NIH Image J version 1.47, http://rsbweb.nih.gov/ij/).

**Figure 2:**
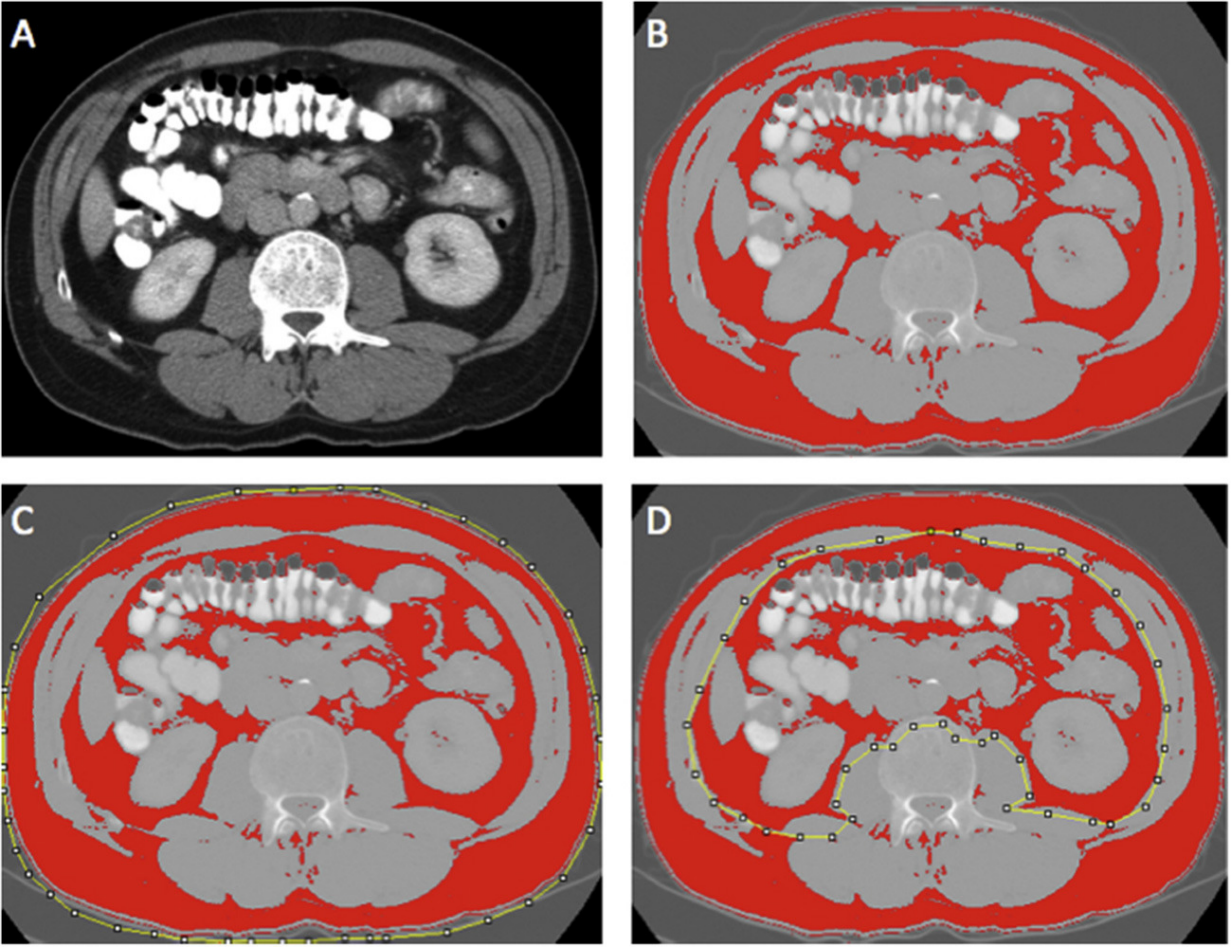
Example of selection of CT body composition—fat areas using ImageJ software; **(A)** mid-L3 vertebra axial slice from preoperative portal venous phase CT, **(B)** threshold selection of adipose tissue using automatic selection of pixels of radiodensity ranging −190 to −30 Hounsfield units (HU); **(C)** region of interest (ROI) selection for total fat area (TFA, cm^2^); and **(D)** ROI selection for visceral fat area (VFA, cm^2^). Adapted from McSorley et al. (22).

**Figure 3:**
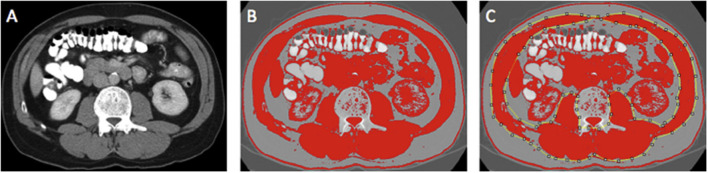
Example of selection of CT body composition—skeletal muscle area (SMA) using ImageJ software; **(A)** mid-L3 vertebra axial slice from preoperative portal venous phase CT; **(B)** threshold selection of skeletal muscle tissue using automatic selection of pixels of radiodensity ranging _29–150 Hounsfield units (HU); **(C)** region of interest (ROI) selection for SMA (cm^2^). Adapted from McSorley et al. (22).

Region of interest measurements of the visceral fat, subcutaneous fat, and skeletal muscle areas (cm^2^) were taken using the standard Hounsfield Unit (HU) ranges (adipose tissue −190 to −30 and skeletal muscle −29 to +150; Figures 2, 3). These were then normalized for height (m^2^) to create the indices—subcutaneous fat index (SFI, cm^2^/m^2^) and SMI (cm^2^/m^2^). The SMD (HU) was measured from the same ROI used to calculate SMI, as its mean HU. The ROI for subcutaneous fat was the area between skeletal muscle and skin. The ROI for visceral fat was the contents of the visceral cavity. The ROI for the skeletal muscle included the quadratus lumborum, psoas, rectus abdominus, erector spinae muscles, and the internal transverse and external oblique muscle groups.

The SMI (Dolan) and the SMD (Dolan) gender-adjusted thresholds were derived using receiver–operating characteristic curve analysis to determine thresholds associated with overall survival in this population. This was conducted using validated online biomarker cutoff optimization software (21). This resulted in SMI (Dolan) being defined as an SMI <51.2 cm^2^/m^2^ in male patients and SMI <41.9 cm^2^/m^2^ in female patients. This resulted in SMD (Dolan) being defined as <34.1 HU in males and <34.4 HU in females. Previously published thresholds were used for visceral obesity (visceral fat area [VFA] >160 cm^2^ for male patients and >80 cm^2^ for female patients (13, 22). These thresholds were similar but not identical to those published by Doyle et al. (23). A high SFI was defined as ≥50.0 cm^2^m^2^ in males and ≥42.0 cm^2^m^2^ in females (6).

The measurements were performed by two individuals (RD) and (TA) and inter-rater reliability was assessed in a sample of images of 30 patients using inter-class correlation coefficients [ICCC; total fat area (TFA) ICCC = 1.000, subcutaneous fat area (SFA) ICCC = 1.000, VFA ICCC = 1.000, skeletal muscle area (SMA) ICCC = 0.998, and SMD ICCC = 0.972]. Investigators were blind to the patient's demographic and clinico-pathological status.

An autoanalyzer was used to measure serum CRP (mg/L) and albumin (g/L) concentrations (Architect; Abbot Diagnostics, Maidenhead, United Kingdom). The mGPS and NLR were derived as previously described (24). The magnitude of the post-operative systemic inflammatory response was measured using the post-operative Day 4 C-reactive protein (CRP; <150 or >150 mg/L) (25, 26). The BMI measurements and blood tests were not routinely carried out on follow-up.

### Statistical Analysis

Body composition measurements were presented as median and range and compared using paired Wilcoxon tests (13, 22). Categorical variables were analyzed using paired McNemar tests for 2 × 2 tables. The changes in body composition and clinicopathological characteristics were presented as median and range and compared using paired Kruskal–Wallis tests (13, 22). Non-paired categorical variables of the patients were analyzed using χ^2^ test for linear-by-linear association, or χ^2^ test for 2 × 2 tables. Linear logistic regression was used to compare significant variables.

Mortality within 30 days of the index procedure or during the index admission was excluded from subsequent survival analysis (13, 22). The time between the date of surgery and the date of death of any cause was used to define overall survival (OS) (13, 22). Survival data were analyzed using univariate categorical Cox regression and Kaplan–Meier analysis (13, 22).

Missing data were excluded from analysis on a variable-by-variable basis. Two-tailed *p* < 0.05 were considered to be statistically significant (13, 22). Statistical analysis was performed using SPSS software (Version 21.0. SPSS Inc., Chicago, IL, United States) (13, 22).

## Results

In total, we identified 976 patients who underwent potentially curative elective surgery for CRC with initial pre-operative scans. Of those, 89 were excluded due to missing clinicopathological data (*n* = 45), blood tests (*n* = 39), and death within 1 month of surgery (*n* = 5). The remaining 887 patients were those with follow-up scans (*n* = 804) and those without follow-up scans (*n* = 83). Of the 804 patients with follow-up scans, 783 (97%) patients were alive at 1 year and of the 83 patients without follow-up scans, 60 (72%) were alive at 1 year (Figure 1). The clinicopathological characteristics of these two patient groups are compared in Table 1.

**Table 1:** Clinicopathological characteristics of patients undergoing potentially curative resection for colorectal cancer with and without follow-up scans at 1 year.

**Peri-operative clinicopathological characteristics**		**Follow-up scan available *N =* 804**	**Follow-up scan not available *N =* 83**	***P*-value**
Age (years)	≤ 65	292 (36.3)	18 (21.7)	<0.001
	65–74	304 (37.8)	23 (27.7)	
	>74	208 (25.9)	42 (50.6)	
Gender	Female	359 (44.7)	36 (43.4)	0.823
	Male	445 (55.3)	47 (56.6)	
ASA score	1	170 (21.1)	10 (12.0)	0.001
	2	372 (46.3)	30 (36.1)	
	3 and 4	262 (32.6)	43 (51.8)	
mGPS	0	600 (74.6)	63 (75.9)	0.744
	1	91 (11.3)	5 (6.0)	
	2	113 (14.1)	15 (18.1)	
NLR	<3	427 (53.1)	39 (47.0)	0.227
	3–5	247 (30.7)	27 (32.5)	
	>5	130 (16.2)	17 (20.5)	
Surgical approach	Open	490 (60.9)	61 (73.5)	0.025
	Laparoscopic	314 (39.1)	22 (26.5)	
TNM stage	I	192 (23.9)	25 (30.1)	0.558
	II	330 (41.0)	28 (33.7)	
	III	282 (35.1)	30 (36.1)	
Venous invasion	No	334 (41.5)	36 (43.4)	0.812
	Yes	470 (58.5)	47 (56.6)	
Tumor location	Right and transverse	303 (37.7)	38 (45.8)	0.320
	Left	122 (15.2)	9 (10.8)	
	Rectum	345 (42.9)	32 (38.6)	
	Total and subtotal	34 (4.2)	4 (4.8)	
BMI (kg/m^2^) continuous	Median, range	27.20 (14.5–59.3)	25.53 (16.0–55.8)	0.039
BMI (kg/m^2^)	<25	267 (33.2)	37 (44.6)	0.038
	≥25	537 (66.8)	46 (55.4)	
SFI continuous	Median, range	76.90 (3–345)	69.35 (3–356)	0.099
Pre-op High SFI	Normal	153 (19.0)	21 (25.3)	0.161
	Subcutaneous obesity	651 (81.0)	62 (74.7)	
VFA continuous	Median, range	188.86 (6.59–674.38)	181.17 (10.06–523.57)	0.554
Pre-op high visceral obesity	Normal	207 (25.7)	28 (33.7)	0.116
	Visceral obesity	597 (74.3)	55 (66.3)	
SMI continuous	Median, range	45.36 (15.51–134.43)	40.49 (25.85–65.63)	0.001
Pre-op high SMI (Dolan Male/Female)	Normal	364 (45.3)	24 (28.9)	0.004
	Sarcopenia	440 (54.7)	59 (71.1)	
SMD continuous	Median, range	32.30 (5.1–67.90)	29.80 (7.20–45.87)	0.008
Pre-op high SMD (Dolan Male/Female)	Normal	346 (43.0)	23 (27.7)	0.007
	Myosteatosis	458 (57.0)	60 (72.3)	
**Post-operative complications**				
Any complication	No complication	488 (60.7)	47 (56.6)	0.471
	Complication	316 (39.3)	36 (43.4)	
Infective complication	No complication	599 (74.5)	61 (73.5)	0.841
	Complication	205 (25.5)	22 (26.5)	
Day 4 CRP (mg/l) +	<150	435 (65.1)	42 (63.6)	0.810
	>150	233 (34.9)	24 (36.4)	
Length of stay	≤ 7 days	369 (45.9)	32 (38.6)	0.201
	>7 days	435 (54.1)	51 (61.4)	
Overall Survival Rate (1-year)	Alive	783 (97.4)	60 (72.3)	<0.001
	Dead	21 (2.6)	23 (27.7)	

*Body composition measurements were presented as median and range and compared using paired Wilcoxon tests (13, 22). Categorical variables were analyzed using paired McNemar tests for 2 by 2 tables. ASA, American Society of Anaesthesiologists; mGPS, modified Glasgow Prognostic Score; NLR, Neutrophil Lymphocyte Ratio; TNM, tumor node metastasis; BMI, Body mass index; SFI, Subcutaneous Fat Index; VFA, Visceral Fat Area; SMI, Skeletal Muscle Index; SMD, Skeletal Muscle Density; CRP, C-Reactive Protein, +, 668*.

When compared with those patients without follow-up scans (*n* = 83, Table 1), patients with follow-up scans (*n* = 804) were younger (*p* < 0.001), had a lower ASA (*p* < 0.01), underwent laparoscopic surgery (*p* < 0.05), had a higher BMI (*p* < 0.05), a higher SMI (*p* < 0.01), a higher SMD (*p* < 0.01), and a better 1-year survival (*p* < 0.001). In patients with follow-up scans, an elevated mGPS was associated with a lower SMI (*p* < 0.001, result not tabulated).

Longitudinal changes in body composition are shown in Table 2 and **Figures 5**–**7**. When the change in SFI was taken as a continuous variable, there was an increase in the median level of 5.60 cm^2^/m^2^ (−158.36 cm^2^/m^2^ to +116.55 cm^2^/m^2^, *p* < 0.001) such that there was a significant increase in the number of patients with an elevated SFI (84 vs. 81%, *p* < 0.05). On Cox regression when taken as a continuous variable, a change in SFI was associated with an improved overall survival (*p* < 0.05). On Cox regression when taken as a categorical variable, a change in SFI was associated with an improved overall survival (*p* < 0.001). On Cox regression when censored for all cases with <1 year of follow up after the second CT, SFI as a categorical variable was associated with an improved overall survival.

**Table 2:** The longitudinal changes in CT-derived body composition measures in patients undergoing surgery for colorectal cancer.

**Body composition** **measurement in** **total cohort**	**Initial CT** **scan (*n =* 804)**	**Follow-up CT** **scan (*n =* 804)**	***P*-Value**	**Changes in** **median**	**Overall survival** **HR 95%CI**	***P*-value**	**Censored Overall** survival* HR **95%CI**	***P*-value**
**Fat**								
SFI	Median (Range) 76.90 (3.00–345.00)	Median (Range) 81.61 (3.07–306.78)	<0.001	Median (Range) 5.60 (−158.36 to +116.55)	0.99 (0.98–1.00)	0.030	1.00 (0.99–1.00)	0.175
High SFI (6)Males>50.0 cm^2^m^2^ and Females>42.0 cm^2^m^2^	Normal: 153 (19.0%) High SFI: 651 (81.0%)	Normal: 128 (15.9%) High SFI: 676 (84.1%)	0.019	High SFI: +3.1%	0.78 (0.69–0.89)	<0.001	0.83 (0.73–0.94)	0.004
VFA	Median (Range) 189.60 (6.59–674.38)	Median (Range) 181.43 (4.61–557.04)	<0.001	Median (Range) −7.36 (−320.46 to +398.75)	1.00 (0.99–1.01)	0.152	1.00 (0.99–1.00)	0.472
High VFA (27, 28)VFA in Males >160 cm^2^ and Females >80 cm^2^	Normal: 207 (25.7%) High VFA: 597 (74.3%)	Normal: 217 (27.0%) High VFA: 587 (73.0%)	0.399	High VFA: −1.3%	0.87 (0.78–0.98)	0.016	0.94 (0.83–1.06)	0.285
**Muscle**								
SMI	Median (Range) 45.36 (15.51-86.70)	Median (Range) 46.83 (18.14–86.70)	<0.001	Median (Range) 1.55 (−80.75 to +36.70)	0.98 (0.96–0.99)	0.024	1.00 (0.98–1.02)	0.954
High SMI (Dolan Male/Female) (13): SMI in Males>51.2 cm^2^m^2^ and Females>41.9 cm^2^m^2^	High SMI: 364 (45.3%) Low SMI: 440 (54.7%)	High SMI: 431 (53.6%) Low SMI: 373 (46.4%)	<0.001	High SMI: +8.3	0.80 (0.72–0.89)	<0.001	0.85 (0.76–0.95)	0.004
SMD	Median (Range) 32.30 (5.10–67.90)	Median (Range) 31.87 (7.30–56.21)	0.062	Median (Range) −0.66 (−34.99 to +27.30)	0.99 (0.97–1.01)	0.175	1.00 (0.98–1.02)	0.645
High SMD (Dolan Male/Female) (13): SMD in Males>34.1 HU and Females>HU 34.4 HU	High SMD: 346 (43.0%) Low SMD: 458 (57.0%)	High SMD: 329 (40.9%) Low SMD: 475 (59.1%)	0.280	High SMD: −2.1%	0.83 (0.74–0.94)	0.002	0.90 (0.80–1.02)	0.097

*Body composition measurements were presented as median and range and compared using paired Wilcoxon tests. Categorical variables were analyzed using paired McNemar tests for 2 by 2 tables. Changes in body composition and clinicopathological characteristics were presented as median and ranges and compared using paired Kruskal–Wallis tests. Survival data were analyzed using univariate categorical Cox regression. SFI, Subcutaneous Fat Index; VFA, Visceral Fat Area; SMI, Skeletal Muscle Index; SMD, Skeletal Muscle Density; CRP, C-reactive protein*.

When the change in VFA was taken as a continuous variable, there was a decrease in the median level of −7.36 cm^2^/m^2^ (−320.46 cm^2^/m^2^ to +398.75 cm^2^/m^2^, *p* < 0.001), but there was a non-significant difference in the number of patients with a high VFA (73 vs. 74%, *p* = 0.399). On Cox regression when taken as a categorical variable, a change in VFA was associated with an improved overall survival (*p* < 0.05). On Cox regression when censored for all cases with <1 year of follow up after the second CT, no measurement of VFA was associated with an improved overall survival.

When the change in SMI was taken as a continuous variable, there was an increase in the median level of 1.55 cm^2^/m^2^ (−80.75 cm^2^/m^2^ to +36.70 cm^2^/m^2^, *p* < 0.001) such that there was a significant decrease in the number of patients with a low SMI (45 vs. 54%, *p* < 0.001). On Cox regression when taken as a continuous variable, an increase in SMI was associated with a better overall survival (*p* < 0.05). On Cox regression when taken as a categorical variable, an increase in SMI was associated with a better overall survival (*p* < 0.001). On Cox regression when censored for all cases with <1 year of follow up after the second CT, SMI as a categorical variable was associated with an improved overall survival.

When the change in SMD was taken as a continuous variable, there was no significant decrease in the median level of −0.66 HU (-34.99 HU to +27.30 HU, *p* = 0.062) such that there was no significant increase in the number of patients with a high SMD (43 vs. 41%, *p* = 0.280). On Cox regression when taken as a categorical variable, an increase in SMD was associated with a better overall survival (*p* < 0.01). On Cox regression when censored for all cases with <1 year of follow up after the second CT, no measurement of SMD was associated with an improved overall survival.

Waterfall plot analysis in **Figures 5**–**7** shows that the longitudinal changes in SFI were associated with mGPS (*p* < 0.001), the longitudinal changes in VFA were associated with gender (*p* < 0.05), and the longitudinal changes in SMI were associated with mGPS (*p* < 0.01).

When linear regression analysis was carried out for the change in SMI against baseline clinicopathological characteristics, including age, gender, BMI, ASA, mGPS, NLR, TNM stage, and venous-invasion only mGPS (*r* = 0.658, *p* = 0.042) and NLR (*r* = 0.524, *p* = 0.084) were significantly associated with the change in SMI (Table 3).

**Table 3:** Linear regression analysis of changes in SMI against baseline clinicopathological characteristic in patients undergoing surgery for colorectal cancer.

**Variable**	**Coefficient B**	***t***	***P*-value**	**95% CI for B**	**Adjusted *R^**2**^***
Age (years)	0.293	1.014	0.311	−0.275 to 0.861	0.001
Gender	0.273	0.600	0.549	−0.620 to 1.166	<0.001
ASA score	0.211	0.675	0.500	−0.402 to 0.824	0.001
mGPS	0.638	2.040	0.042	0.024 to 1.253	0.005
NLR	0.524	1.731	0.084	−0.070 to 1.118	0.004
TNM stage	0.215	0.723	0.470	−0.369 to 0.800	−0.001
Venous invasion	0.052	0.113	0.910	−0.849 to 0.953	<0.001
BMI≥25	0.115	0.240	0.811	−0.828 to 1.058	<0.001

**Figure 4:**
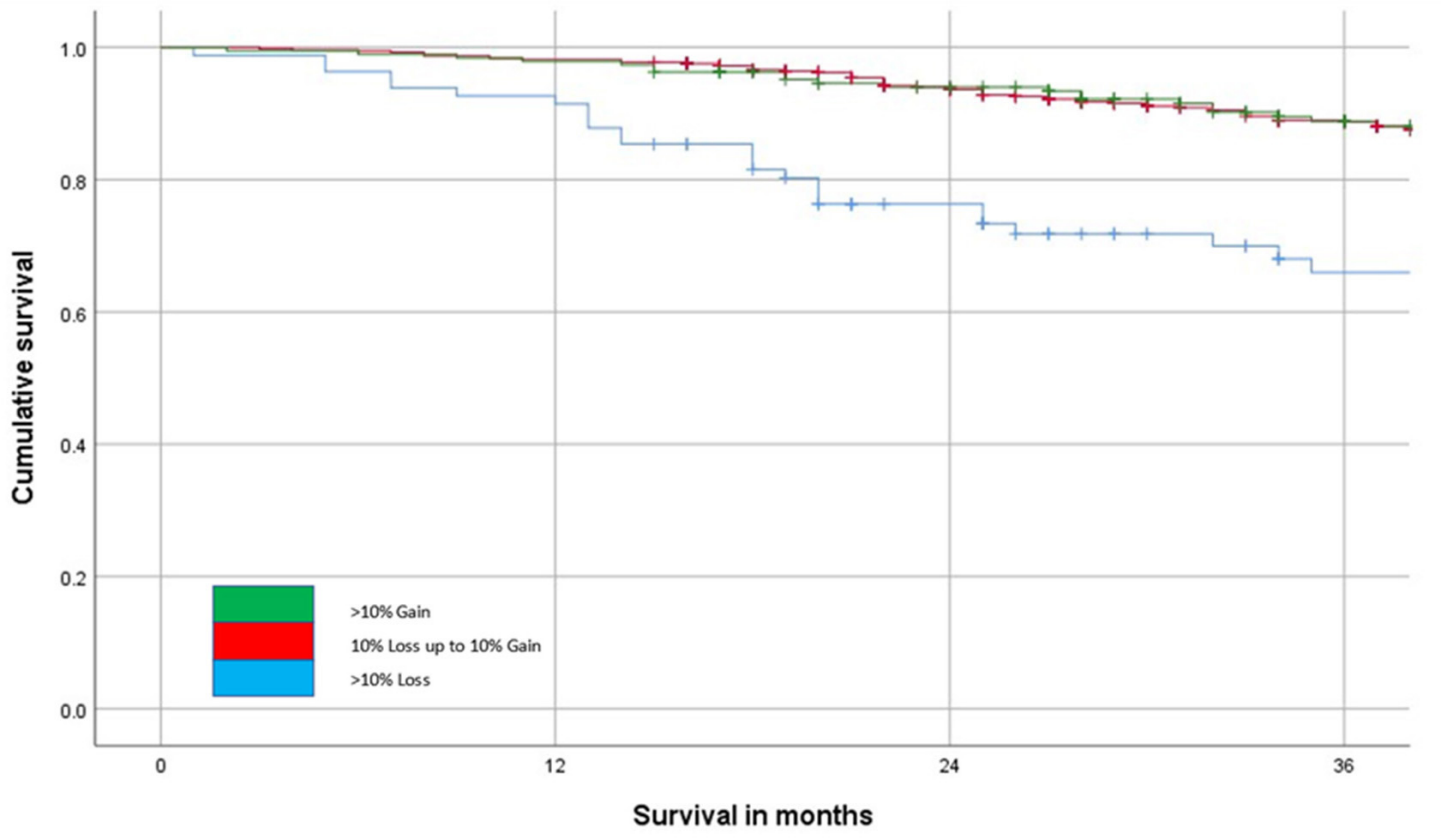
The relationship between change in SMI on follow-up and overall survival (*n* = 804, *p* < 0.001) over a 36-month follow-up period.

The relationship between the change in SMI (>10% loss or <10% loss to <10% gain or >10% gain) and overall survival is shown in Figure 4. This shows that patients that lose in excess of 10% of their SMI have poorer outcomes when compared with patients that lose <10% or indeed gain SMI on follow-up (*p* < 0.001).

## Discussion

**Figure 5:**
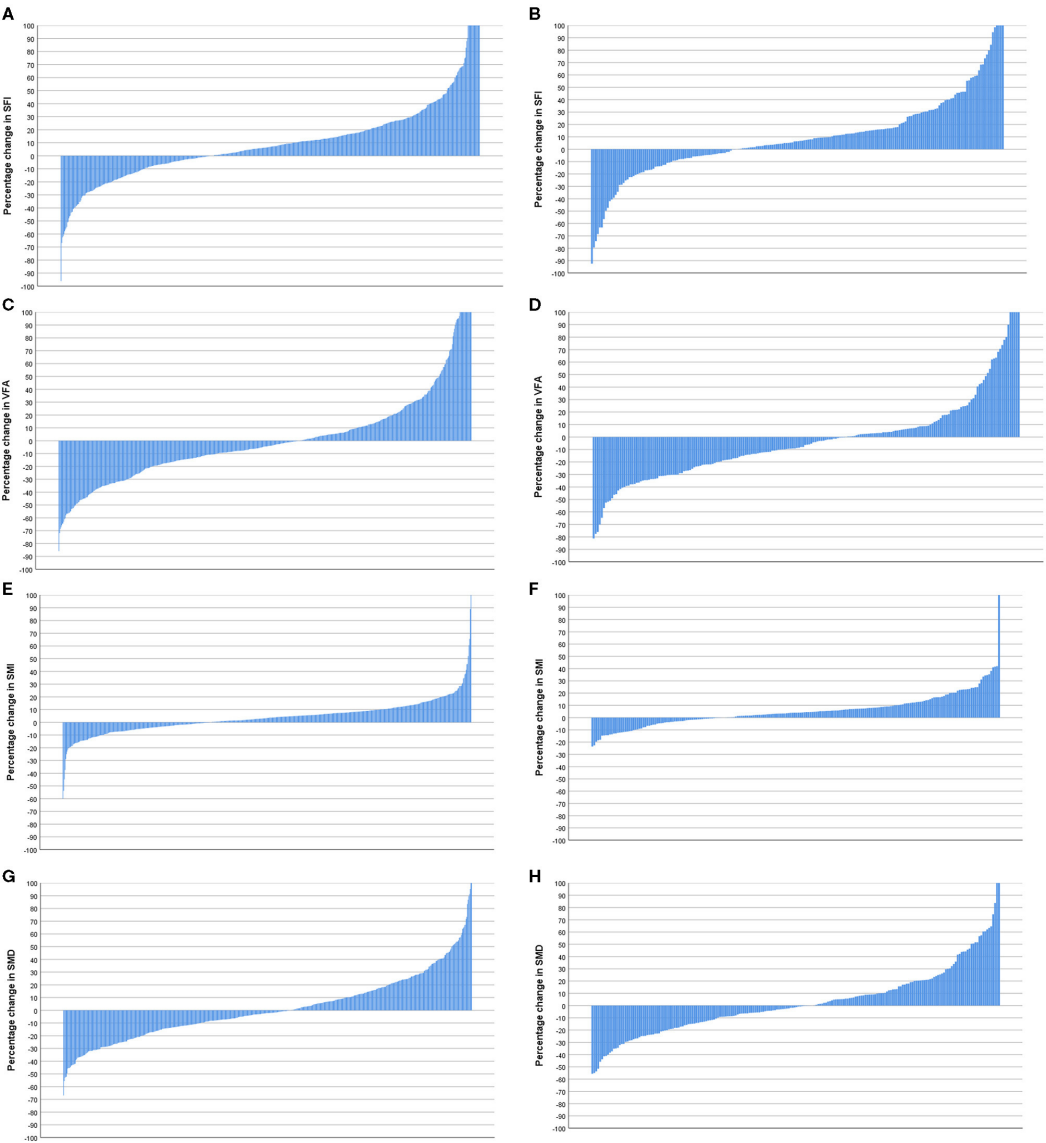
**(A)** Percentage change in subcutaneous fat index (SFI) in patients <74 years of age (*n* = 596). **(B)** Percentage change in SFI in patients >74 years of age (*n* = 208). Comparison of percentage change in SFI in patients <74 years (*n* = 596) and >74 years of age (*n* = 208) (*p* = 0.922). **(C)** Percentage change in visceral fat area (VFA) in patients <74 years of age (*n* = 596). **(D)** Percentage change in VFA in patients >74 years of age (*n* = 208). Comparison of percentage change in VFA in patients <74 years (*n* = 596) and >74 years of age (*n* = 208) (*p* = 0.171). **(E)** Percentage change in skeletal muscle index (SMI) in patients <74 years of age (*n* = 596). **(F)** Percentage change in SMI in patients >74 years of age (*n* = 208). Comparison of percentage change in SMI in patients <74 (*n* = 596) and >74 years of age (*n* = 208) (*p* = 0.197). **(G)** Percentage change in skeletal muscle density (SMD) in patients <74 years of age (*n* = 596). **(H)** Percentage change in SMD in patients >74 years of age (*n* = 208). Comparison of percentage change in SMI in patients <74 years (*n* = 596) and >74 years of age (*n* = 208) (*p* = 0.721).

**Figure 6:**
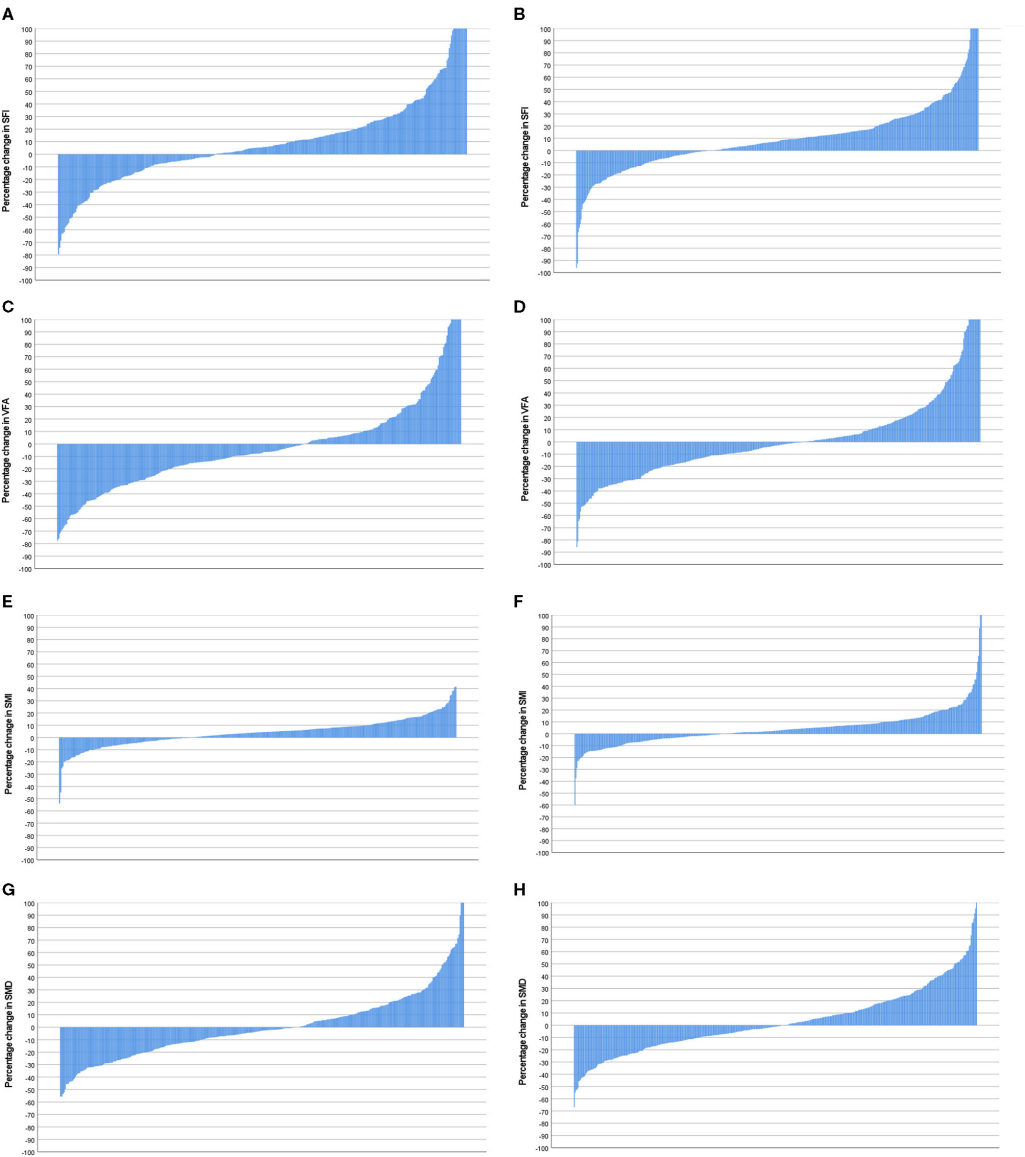
**(A)** Percentage change in SFI in female patients (*n* = 359). **(B)** Percentage change in SFI in male patients (*n* = 445). Comparison of percentage change in SFI in female (*n* = 359) and male (*n* = 445) patients (*p* = 0.380). **(C)** Percentage change in VFA in female patients (*n* = 359). **(D)** Percentage change in VFA in male patients (*n* = 445). Comparison of percentage change in VFA in female (*n* = 359) and male (*n* = 445) patients (*p* = 0.04). **(E)** Percentage change in SMI in female patients (*n* = 359). **(F)** Percentage change in SMI in male patients (*n* = 445). Comparison of percentage change in SMI in female (*n* = 359) and male (*n* = 445) patients (*p* = 0.324). **(G)** Percentage change in SMD in female patients (*n* = 359). **(H)** Percentage change in SMD in male patients (*n* = 445). Comparison of percentage change in SFD in female (*n* = 359) and male (*n* = 445) patients (*p* = 0.089).

**Figure 7:**
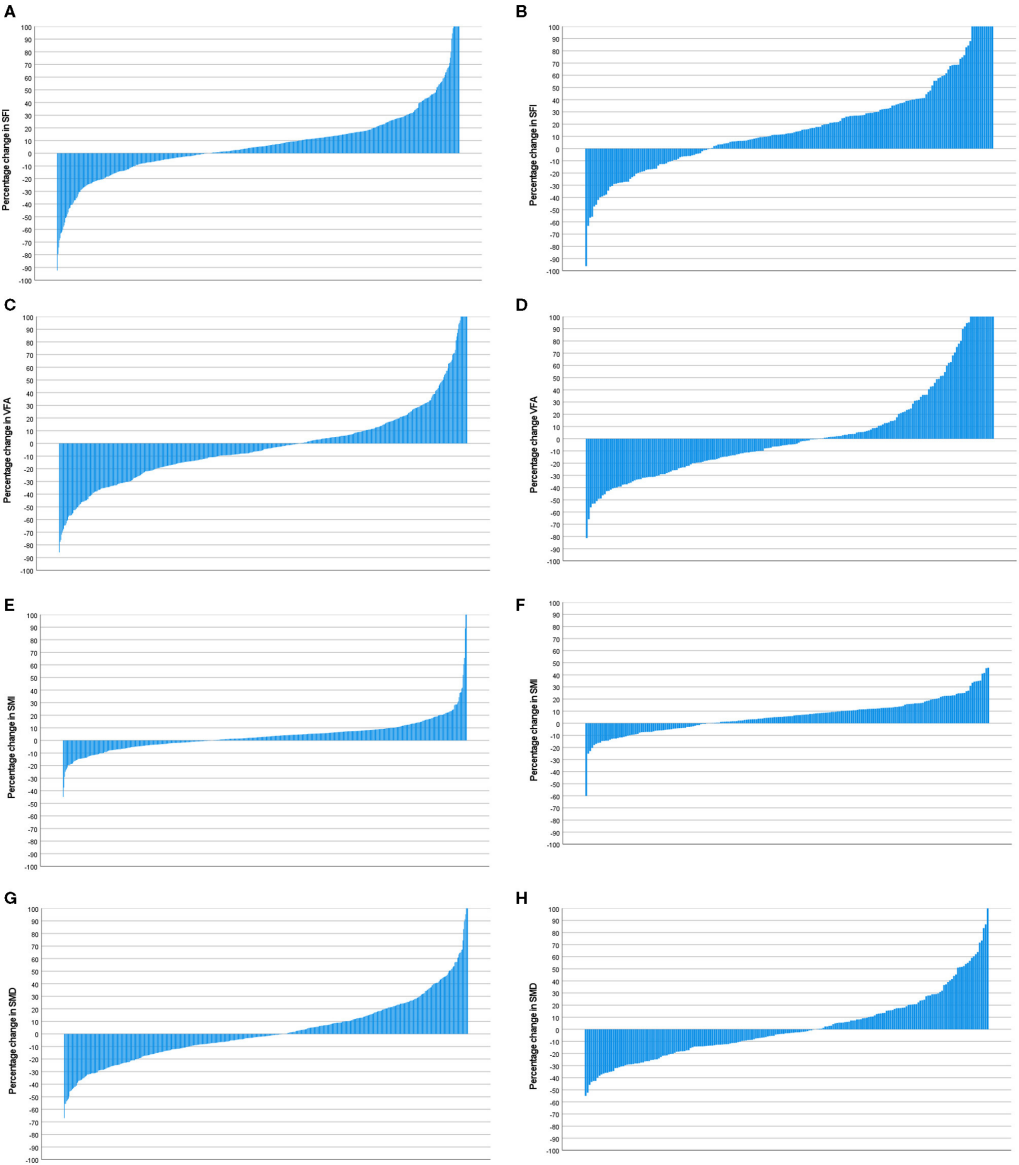
**(A)** Percentage change in SFI in patients with a mGPS 0 (*n* = 600). **(B)** Percentage change in SFI in patients with a mGPS 1 or 2 (*n* = 204). Comparison of percentage change in SFI in mGPS 0 (*n* = 600) and mGPS 1 or 2 (*n* = 204) patients (*p* < 0.001). **(C)** Percentage change in VFA in patients with a mGPS 0 (*n* = 600). **(D)** Percentage change in VFA in patients with a mGPS 1 or 2 (*n* = 204). Comparison of percentage change in VFA in mGPS 0 (*n* = 600) and mGPS 1 or 2 (*n* = 204) patients (*p* = 0.614). **(E)** Percentage change in SMI in patients with a mGPS 0 (*n* = 600). **(F)** Percentage change in SMI in patients with a mGPS 1 or 2 (*n* = 204). Comparison of percentage change in SMI in mGPS 0 (*n* = 600) and mGPS 1 or 2 (*n* = 204) patients (*p* = 0.002). **(G)** Percentage change in SMD in patients with a mGPS 0 (*n* = 600). **(H)** Percentage change in SMD in patients with a mGPS 1 or 2 (*n* = 204). Comparison of percentage change in SMD in mGPS 0 (*n* = 600) and mGPS 1 or 2 (*n* = 204) patients (*p* = 0.289).

Given the almost universal prognostic value of pre-treatment CT-based measurements of sarcopenia (low SMI), there is a considerable interest in therapeutic targeting of SMI. However, to date, a few longitudinal studies have been carried out to test this relationship. The results of the present longitudinal study showed that in 47 (5.8%) patients there was a reduction in SMI and in 114 (14.2%) patients there was an increase in SMI. Therefore, only ~20% of the patients showed changes in their SMI (*n* = 161) and even a lesser number of patients showed relative changes of 10% or more (see Figures 4–7). In the largest longitudinal study to date, Brown and co-workers carried out such analysis using two standard deviations from the mean change as an indication of a significant loss or gain of SMI and related this to survival (29). The loss of SMI (but not the gain of SMI) was significantly associated with survival and applied to a small proportion of the population studied (~7%) and therefore the clinical value of longitudinal CT-derived body composition analysis in patients undergoing surgery for primary CRC would appear limited.

These observations have a number of implications. First, they would suggest that, since SMI is relatively stable over at least 12 months (Table 2, see Figures 4–7), the majority of losses in SMI occur before the diagnosis and therefore are likely to be largely constitutional (i.e., the die is cast at an early stage in the cancer journey) rather than as a result of cancer itself. Moreover, as a result, it is likely that most of the prognostic values can be derived from the initial body composition measurements compared with the follow-up measurements in primary operable colorectal cancer. Second, the consistent association, in both cross-sectional (15) and now in longitudinal studies between a low SMI and the mGPS may suggest a causal relationship. If these were causally linked, then it might be expected that changes in SMI status would be associated with changes in systemic inflammatory status. Although there is abundant evidence that the systemic inflammatory response is associated with profound catabolism of skeletal muscle and may also block anabolism, a few studies have attempted to target directly the systemic inflammatory response and monitor skeletal muscle mass in patients with either primary operable cancer or in advanced inoperable cancer (15).

On linear regression analysis against baseline clinicopathological characteristic only mGPS (*r* = 0.658, *p* = 0.042) and NLR (*r* = 0.524, *p* = 0.084) were significantly associated with a change in SMI. This is consistent with the cross-sectional studies that have shown a consistent association between the systemic inflammatory response and SMI in patients with cancer (15).

In the present study to examine longitudinal changes in body composition follow-up, CT scans were taken at ~12 months after surgery for colorectal cancer, in line with the current follow-up protocols. As a consequence, the large majority of patients had a follow-up body composition measurement (i.e., 804 of 1,047 patients) at the same time point and also had a comprehensive examination of potentially important factors in body composition change. In contrast, Malietzis et al. from an initial cohort of 1,477 patients with colorectal cancer, examined multiple follow-up scans at different time points (2,136 scans in 856 patients) and modeled these to give changes in SMI over time (18). Similarly, Brown and co-workers, from an initial cohort of 3,262 patients with colorectal cancer, examined a follow-up scan 9–27 months after diagnosis in 1,924 patients and examined changes in SMI over time (29). Finally, Hopkins et al. from an initial cohort of 1,418 patients with colorectal cancer, examined a follow-up scan 24 months after diagnosis in 667 patients and examined changes in SMI over time (30). Therefore, compared with these previous studies, the present study is likely to accurately reflect the changes in body composition that occur in the routine clinical scenario and the associations that are potentially important in changes in body composition.

In a recent paper, Martin et al. published age- and gender-specific thresholds for patients undergoing surgery for CRC (*n* = 2,100). When similar stratification was applied to the present cohort, the results for skeletal muscle volume and radiodensity were comparable despite that there was more advanced disease in the combined Canadian and UK cohorts. However, in the present cohort, there was a greater level of both visceral and subcutaneous fat. This is perhaps not surprising given the deprivation levels of patients referred to Glasgow Royal Infirmary. Indeed, in Glasgow, 190,000 or just under 32% of the city's population resides in the 10% of the most deprived areas of the United Kingdom (the so-called “Glasgow effect”). This is associated with a poor-quality diet, low physical fitness, and high levels of alcohol consumption and smoking, which would have a direct effect on adiposity and comorbidity.

With reference to delineating the relationship between longitudinal changes in CT-derived body composition, clinicopathological characteristics, and the systemic inflammatory response, it may have been better to examine these relationships in patients with advanced cancer since the rate of loss of body tissue is likely to be higher. However, such longitudinal studies are few. For example, McMillan and co-workers reported that in a longitudinal study of 18 male patients- with advanced cancer, those with an elevated CRP concentration lost body cell mass (using a total body potassium counter) at a higher rate (31). Wallengren et al. reported that, in a longitudinal study of 471 patients with advanced cancer, those patients with an elevated CRP concentration had less muscle mass (using dual energy X-ray absorptiometry) on study entry and lost muscle mass at an accelerated rate during follow-up, particularly in males (32). More recently, Huang et al. (33) reported that, in 139 patients with advanced ovarian cancer, there was an average SMI loss of 2% over 6 months and was significantly associated with the mGPS (33). Moreover, pre-treatment SMI and SMI change were independently associated with overall survival. Taken together the present and previous results indicate that both SMI and the mGPS are clinically useful measurements during the treatment of patients with cancer.

## Limitations

The limitations of the present study include its retrospective nature and those only patients with an electronically available CT scan were included in the analysis. Moreover, not all patients had follow-up CT scans at ~12 months and those patients who did not have a follow-up CT scan were older, had a higher ASA, had a lower BMI, lower SMI, lower SMD, and higher mortality. Finally, in those patients with follow-up scans, the median follow-up was <36 months (30.1 months), and therefore only 1-year survival rates were commented on. However, the study population was relatively large, most patients had follow-up scans and were well-documented in terms of clinicopathological characteristics, body composition, and measures of the systemic inflammatory response.

## Conclusions

The present longitudinal study provides further evidence that low–skeletal muscle mass is associated with the presence of a systemic inflammatory response and new evidence that this relationship is established early in the disease course, maintained following resection of the primary tumor in patients with colorectal cancer. Intervention studies are required to establish whether the relationship between low–skeletal muscle mass and the systemic inflammatory response is causal in nature.

## Data Availability Statement

The raw data supporting the conclusions of this article will be made available by the authors upon request, without undue reservation.

## Ethics Statement

The studies involving human participants were reviewed and approved by Ethical approval was granted by the West of Scotland Research Ethics Committee, Glasgow. All research was performed in accordance with the Declaration of Helsinki. Consent for inclusion within clinical research is take at the time of resection. Written informed consent for participation was not required for this study in accordance with the national legislation and the institutional requirements.

## Author Contributions

RD: study conceptualization, scanning and data analysis, and drafting of the manuscript. TA: scanning and data analysis and drafting of the manuscript. WS, AA, and LD: data analysis and drafting of the manuscript. PH, SM, and DM: supervision and editing of the manuscript. All authors contributed to the article and approved the submitted version.

## Conflict of Interest

The authors declare that the research was conducted in the absence of any commercial or financial relationships that could be construed as a potential conflict of interest.

## Publisher's Note

All claims expressed in this article are solely those of the authors and do not necessarily represent those of their affiliated organizations, or those of the publisher, the editors and the reviewers. Any product that may be evaluated in this article, or claim that may be made by its manufacturer, is not guaranteed or endorsed by the publisher.
